# Study on the optimal position of the roof low roadway based on the response surface methodology

**DOI:** 10.1038/s41598-021-93997-w

**Published:** 2021-07-15

**Authors:** Hongqing Zhu, Shuhao Fang, Yujia Huo, Qi Liao, Lintao Hu, Yilong Zhang, Feng Li

**Affiliations:** 1 School of Emergency Management and Safety Engineering, China University of Mining and Technology (Beijing), Beijing, 100083 China; 2 State Key Laboratory Coal Resources and Safe Mining, China University of Mining and Technology (Beijing), Beijing, 100083 China

**Keywords:** Natural hazards, Coal

## Abstract

For determine the optimum position of the roof low roadway, the optimal solution is derived according to the response surface methodology. The UDEC numerical simulation of the overburden gives the porosity distribution of the strike fractured zone, the upper limit heights of the caving zone and the fractured zone are obtained as 18 m and 65 m, respectively. Based on the porosity distribution, the FLUENT numerical models of the goaf zone, air inlet roadway, air return roadway, working face and roof low roadway were established to simulate the gas concentration in the upper corner and gas drainage volume in roof low roadway during mining. Using the vertical and horizontal distance of the roof low roadway as the influencing factors, the experimental scheme of the position of the roof low roadway was designed according to the response surface method, and the response values were obtained from the FLUENT simulation experiments, predicting that the vertical and horizontal distances of the roof low roadway were 7.7 m and 5.9 m respectively when the interaction between the gas concentration in the upper corner and gas drainage volume in roof low roadway was optimal. Field tests showed that the average gas concentration in the upper corner and the average gas drainage volume in roof low roadway were 0.432% and 40.861 m^3^/min respectively, both of which were less than 10% of the error from the simulations. The design of the roof low roadway has effectively managed the gas accumulation problem in the upper corner.

## Introduction

In China, gas hazards are still a prominent problem and gas accidents are often accompanied by loss of life^1–5^. Gas in the upper corner of the working face is a common gas problem and is managed in a number of ways^6,7^. Li et al.^8^ concluded that Y-type ventilation can reduce gas concentration in the upper corner. Xie^9^, Gao^10^ and Yang^11^ et al. concluded that directional long drilling group could effectively manage the gas in the upper corner. Lu et al.^12^ investigates the effectiveness of four gas drainage methods (high drill holes drainage, buried pipe drainage, adjacent roadway drainage and tail roadway drainage)^13,14^ for gas control in air return roadway. Skotniczny^15^ and Guo^16^ et al. studied the transport pattern of gas from the upper corner. Li et al.^17^ investigated methods for managing gas in the upper corner at different gas outflow levels. Wang et al.^18,19^ studied the methane distribution at a longwall working face.

Liu et al.^20^ used FLUENT software to simulate the effect of ground drilling location on gas drainage on the working face. Wang et al.^21^ used FLUENT software to simulate the air flow effect in the goaf, based on the porosity of the overlying rock obtained by PFC simulation. Brodny et al.^22^ used FLUENT to numerically simulate the impact of crushed rock types in the goaf on the air flow in the goaf. Zhang et al.^23^ conducted FLUENT numerical simulation of the dust distribution on the footway in the goaf. Chen et al.^24^ used FLUENT software to study the law of air movement in the return air roadway. Zhou et al.^25^ used FLUENT software to study the distribution of various gases in the goaf. Deng et al.^26^ used FLUENT software to study the influence of gas drainage on the distribution of oxidation zones in the goaf.

Cao^27^ and Zhang^28^ et al. investigated the gas drainage from the dug-in coal seam in the floor roadway; however, the floor roadway could not extract gas from the upper corner during mining. Li et al.^29^ studied the gas drainage from adjacent workings sharing a roof high roadway. Tang et al.^30^ conclude that gas drainage from roof high roadway has negative effect on the controlling the air leakage into the goaf. Zhang et al.^31^ investigated the effect of the position of roof high roadway on gas management. The roof high roadway is not effective in extracting gas from the upper corner, so it is necessary to design a roof low roadway to address the gas in the upper corner^32,33^. Gao et al.^34^ analysed the stress distribution in the surrounding rocks of the roof low roadway. Zheng et al.^35^ concluded that tensile failure occurs mainly on the upper and lower sides of the roadway, while shear failure symmetrically occurs on the left and right sides.

There is relatively little research related to roof low roadway. The optimal position of the roof low roadway is investigated, which can be used to pre-extraction gas from this seam to cover the coal roadway excavation, and also to extraction gas from the upper corner during mining. The experimental scheme is designed according to the response surface methodology^36–39^, and the response values are derived from the FLUENT numerical simulation experiments, and the optimal solution is predicted. The design of the roof low roadway has effectively managed the gas accumulation problem in the upper corner.

## Porosity and roof low roadway

### Porosity of the goaf zone

The theory based on whether the shear stress reaches the shear strength as the failure criterion is the Moore–Coulomb theory^40–42^. Based on the Mohr–Columb principal structure model in the discrete element UDEC software, a numerical calculation model for overburden strike mining at the 15,106 working face was established, with the model gravitational acceleration set to 9.8 m/s^2^ and the coal seam burial depth of 396 m, resulting in a mean distributed load of 9.5 MPa at the top and a pressure measurement coefficient of 0.8 according to the coal seam burial depth. The model size is 500 m × 120 m, and the overburden parameters are shown in Table 1.

**Table 1:** Overburden parameters.

No.	Lithology	Density (kg/m^3^)	Bulk modulus (Gpa)	Shear modulus (Gpa)	Cohesion (Mpa)	Friction angle (°)	Tensile strength (Mpa)
19	Sandy mudstone	2650	2	13.5	3.2	42	1.2
18	Fine sandstone	1400	2	1.3	2.3	27	2.3
17	No. 9 coal	2660	3.5	2.3	2.1	36	0.7
16	Sandy mudstone	2650	2	13.5	3.2	42	1.2
15	Coarse sandstone	2500	5.7	4.1	5.0	38	0.8
14	K4 Limestone	2650	2.5	1.8	7.1	45	1.5
13	Sandy mudstone	2500	3.5	2.3	2.1	36	0.7
12	Coarse sandstone	2570	9.7	6.1	8.0	40	0.8
11	Mid-stone	2600	5.8	4.3	5.0	38	0.9
10	Sandy mudstone	2400	3.5	2.3	2.1	36	0.7
9	K3 Limestone	2650	2.5	1.8	7.1	45	1.5
8	Fine sandstone	2650	2	13.5	3.2	42	1.2
7	Sandy mudstone	2500	3.5	2.3	2.1	36	0.7
6	K2 Limestone	2650	2.5	1.8	7.1	45	1.5
5	Sandy mudstone	2650	2	1.3	2.1	36	0.7
4	Fine sandstone	2650	2.5	1.8	7.1	45	1.5
3	Sandy mudstone	2500	3.5	2.3	2.1	36	0.7
2	No. 15 coal	1400	2	1.3	2.3	27	2.3
1	Sandy mudstone	2500	3.5	2.3	2.1	36	0.7

**Figure 1:**
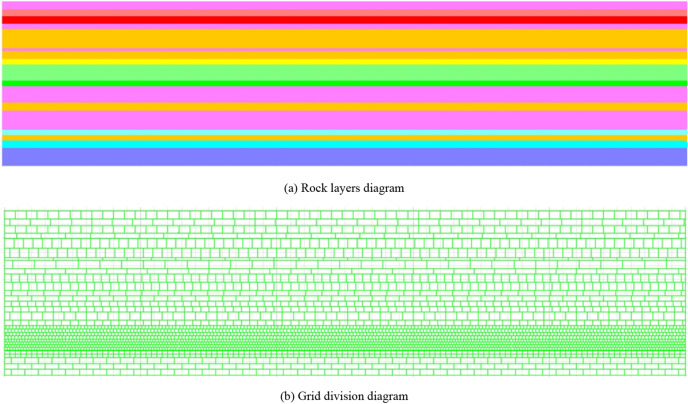
The model (UDEC 6.0).

The rock layers are divided according to the overburden distribution, and the rock layers and grid are shown in Fig. 1a,b. The model was mining 38 times, the first 37 times for 8 m each and the last time for 4 m, for a total mining of 300 m.

**Figure 2:**
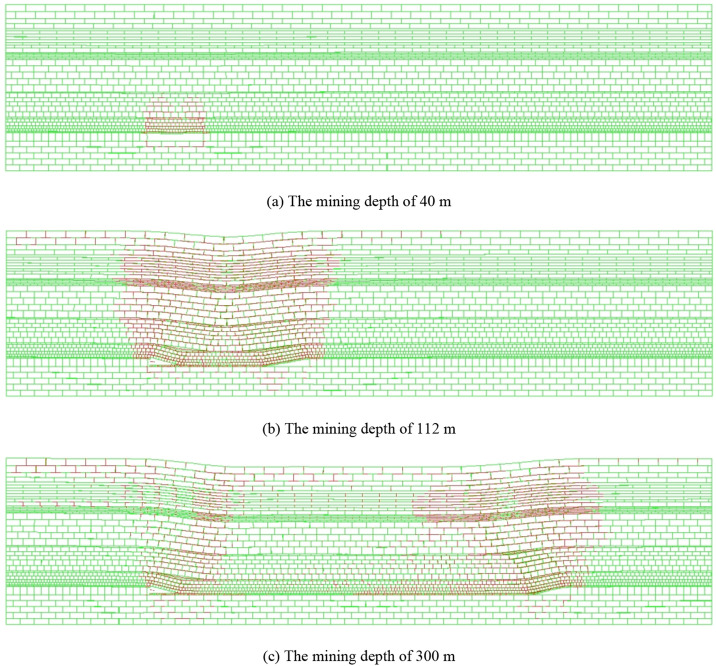
The distribution of overburden fractures (UDEC 6.0).

The distribution of overburden fractures at mining depths of 40 m, 112 m and 300 m is shown in red line in Fig. 2a–c.

The roof of the coal seam had not collapsed and the overburden had developed "arch" type off-bed fractures at the coal mining depth of 40 m. The direct roof of the coal seam had collapsed and the overburden produced a large number of off-bed fractures and through-bed fractures at the coal mining depth of 112 m. The off-bed fractures and through-bed fractures are mainly concentrated on the open-off cut side and mining side due to re-compaction of the overburden at the coal mining depth of 300 m. It can be inferred that the upper limit of the caving zone height is 18 m and the upper limit of the fractured zone height is 65 m, based on the extent of fracture development in the model.

**Figure 3:**
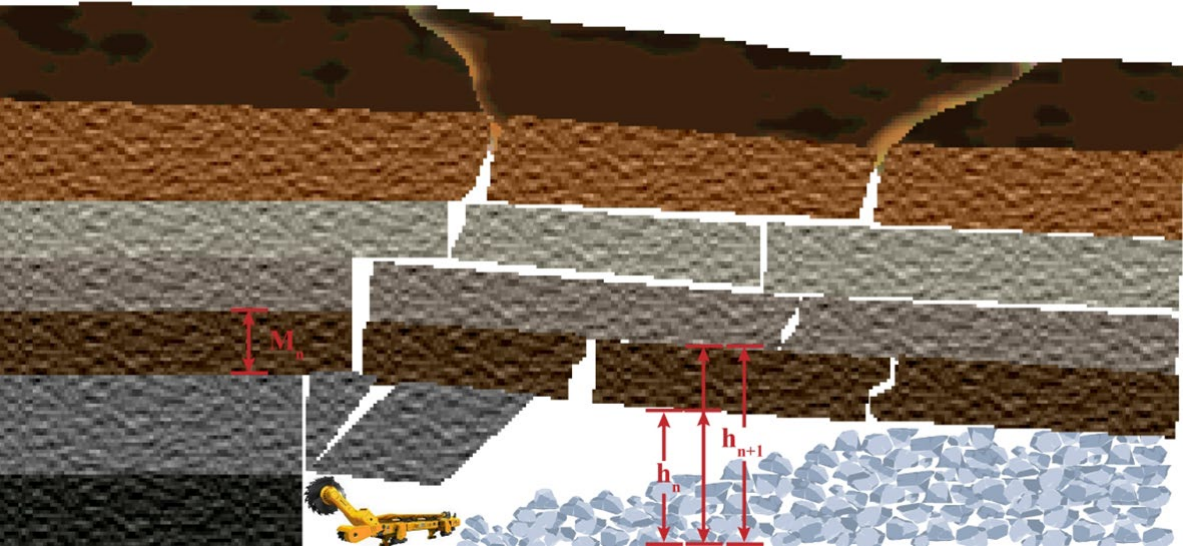
Diagram of overlying porosity (Adobe Illustrator CC 2018).

For the overburden rock in the goaf area, the pore volume of the rock is proportional to the difference between the thickness of the fallen rock and the original rock^43^. The porosity can be expressed as the ratio of the pore volume in the rock body to the total volume of the fallen rock body, as shown in Eq. (1)^44^ and Fig. 3

1 $$p=\frac{{h}_{n+1}-{h}_{n}-{M}_{n}}{{h}_{n+1}-{h}_{n}}$$

where *hn*+*1* is the height of the upper intersection of the fallen rock, m; *hn* is the height of the lower intersection of the fallen rock, m; *Mn* is the thickness of the original rock, m.

**Figure 4:**
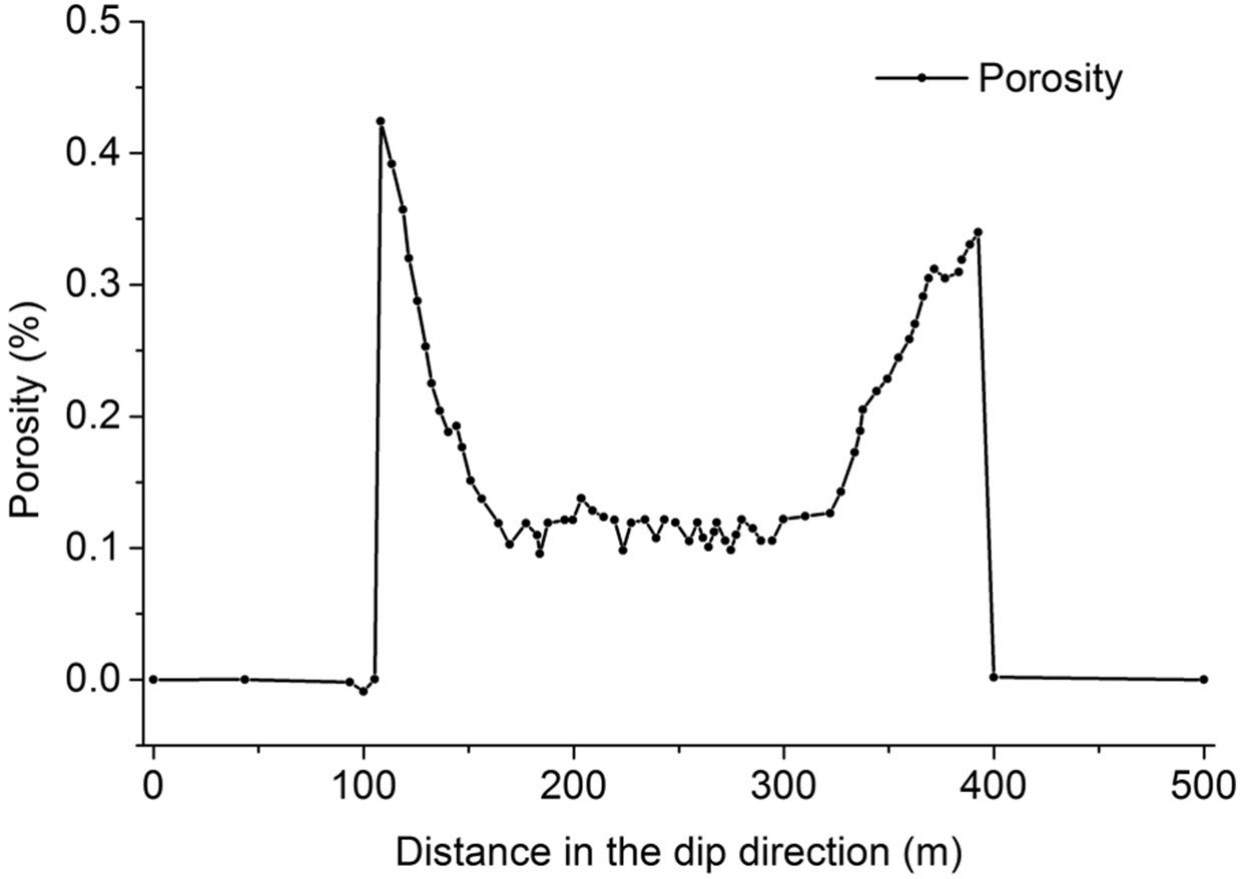
The porosity distribution of the strike caving zone.

Based on the numerical simulation results, the porosity distribution of the strike caving zone is derived according to Eq. (1) and is shown in Fig. 4.

The porosity reaches a maximum of about 0.42 on the open-off cut side and about 0.34 on the mining side, while the porosity in the middle of the goaf is at a relatively small plateau of about 0.1 due to recompaction. Converting two dimensions to three dimensions, the porosity distribution is consistent with the "O" shaped ring theory.

### Range of position in the roof low roadway

The rectangular section of the air return roadway is 4 m high and 5 m wide, the rectangular section of the roof low roadway is 3 m high and 4 m wide. As the mine is an outburst mine, according to the "Detailed rules for prevention of coal and gas outburst", to ensure safety, the vertical distance between the rock roadway and the coal roadway, i.e., the vertical distance, must be greater than 5 m.

**Figure 5:**
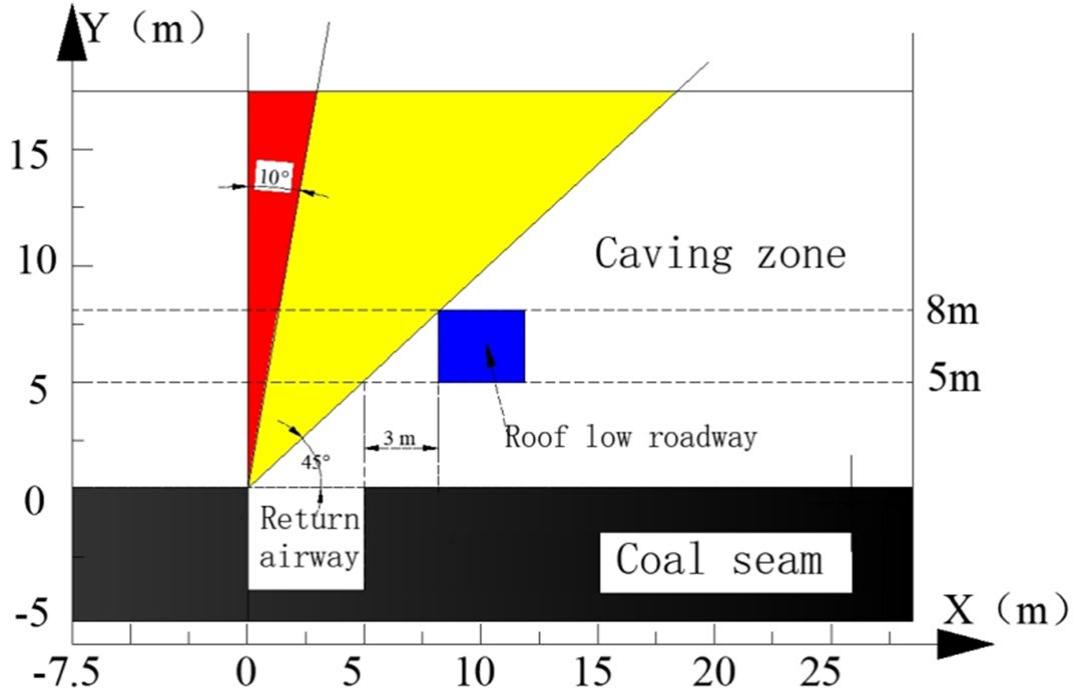
Schematic diagram of the roof low roadway.

The fracture angle of the overlying rock layer is generally 45°–80°^45^. To ensure that the roof low roadway is within the caving zone, the horizontal distance between the roof low roadway and the air return roadway, i.e., the horizontal distance is 3 m when the vertical distance is 5 m. The schematic diagram of the nearest position between the roof low roadway and the air return roadway is shown in Fig. 5.

The yellow area in Fig. 5 is the fracture angle range, and the roof low roadway should be within the 45° fracture angle range. The closer the distance between the roof low roadway and the air return roadway, the better it is for the construction of downward drilling in the roof low road. Taking this into account, the range of position in the roof low roadway is determined as follows: 5–9 m for the vertical distance and 3–7 m for the horizontal distance.

## Fluent numerical simulation

### Goaf model and its gas distribution

Assumptions: (1) The variation in temperature and heat transfer during the flow of the fluid is not taken into account, and the mixture of gas and air is treated as an incompressible gas, ignoring the influence of temperature on the change in volume of the gas. (2) The goaf is regarded as porous medium, and its transverse is divided into natural accumulation zone, stress loading zone and re-compaction zone; In the longitudinal direction, only considering caving zone and fractured zone. The porosity and viscous resistance coefficient of each zone are uniformly distributed and isotropic, but vary from zone to zone.

**Figure 6:**
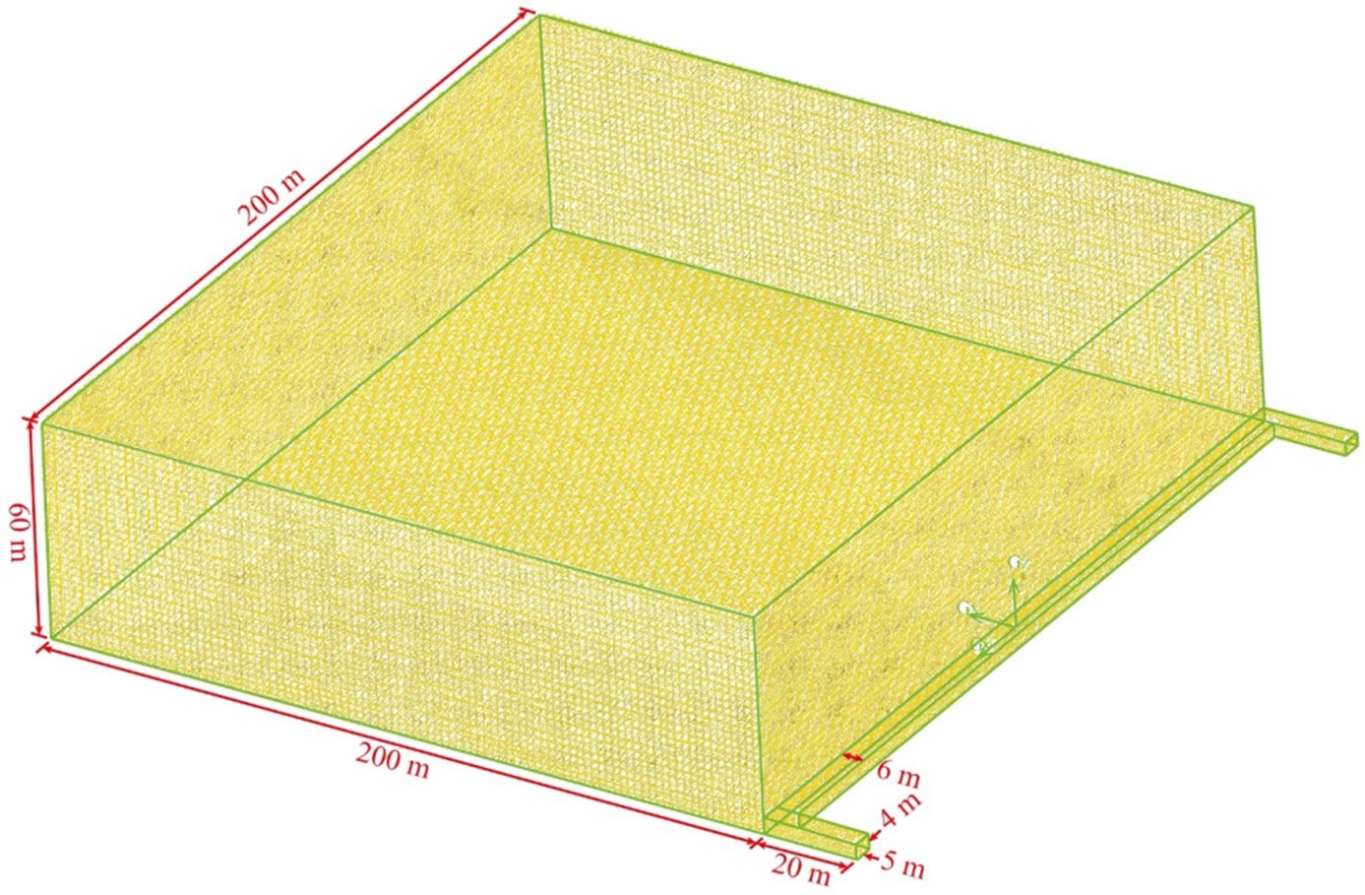
The geometric model (FLUENT 16.0).

Combined with the field situation, the geometric model is established and meshed. The model has an inclination of 6°. The goaf, working face, and roadway models are all regular hexahedrons. Submap type grids can create hexahedral grids, which can ensure calculation accuracy and save time and cost. The mesh type is submap^46^ and 15,446,400 mesh zones are divided, the model is shown in Fig. 6.

The calculation of turbulence intensity is shown in Eq. (2)^47^.

2 $$I=0.16{\left(\frac{\upsilon d\rho }{\mu }\right)}^{-0.125}$$

where *I* is the turbulence intensity; *υ* is the air velocity, m/s; *d* is the hydraulic diameter, m; *ρ* is the air density, 1.29 kg/m^3^; *μ* is the air dynamic viscosity, 1.69×10–6 Pa s.

The standard *k*–*ε* model is selected as the calculation model^48^. The gas component is mixture of methane and air, and the gravity is set at − 9.81 m/s^2^. The entrance of air inlet roadway is set to velocity-inlet. The air velocity measured by the air meter on site is 2.9 m/s. The hydraulic diameter is 4.4 m and the outlet of the air return roadway is set as free outflow. The turbulence intensity calculated by Eq. (2) is 2.14%. Fan is set at the interface between roof roadway and goaf, the negative pressure is 3 kPa, and the outlet of roof roadway is free outflow. The interface between working face and goaf is set as interior, and the interface of roof roadway in goaf is also interior. The other surfaces are set as wall. Each zone is set as fluid zone, and the porous zone, laminar zone and source terms are activated in the goaf. The porous media zone parameters are set using the UDF program and the source option sets the gas mass source item to 2.5 × 10^–7^ kg/m^3^ s.

**Figure 7:**
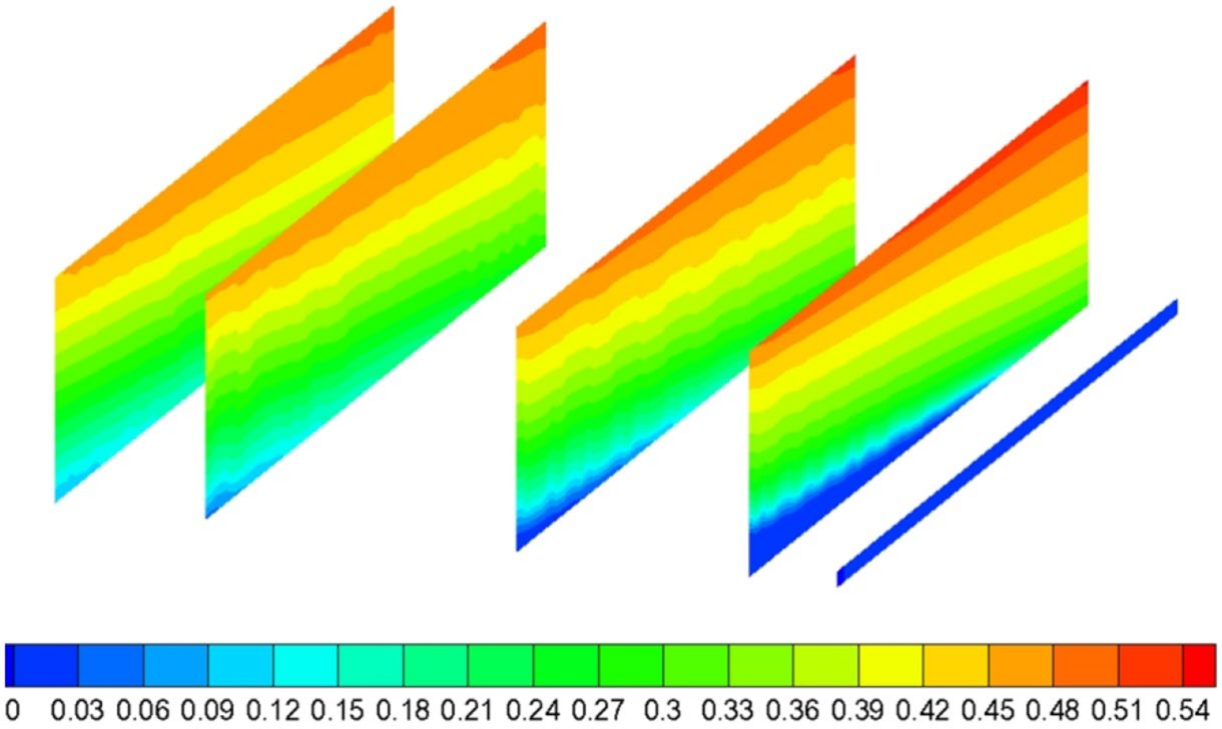
The distribution of gas in the goaf zone (FLUENT 16.0).

The distribution of gas in the goaf zone during normal mining is shown in Fig. 7.

The gas concentration rises gradually as the deepening of goaf in the horizontal direction. In the vertical direction, in the upper part of the extraction area, there is very little air leakage and the gas concentration increases with height under the influence of gas uplift and gravity. On the air inlet roadway side, which is more affected by air leakage, the gas concentration is lower. On the air return roadway side, gas accumulation occurs, with maximum gas concentrations of 28%, 42%, 50% and 56% at 1 m, 20 m, 40 m and 60 m from the floor, respectively.

### Model of roof low roadway and its drainage effect

**Figure 8:**
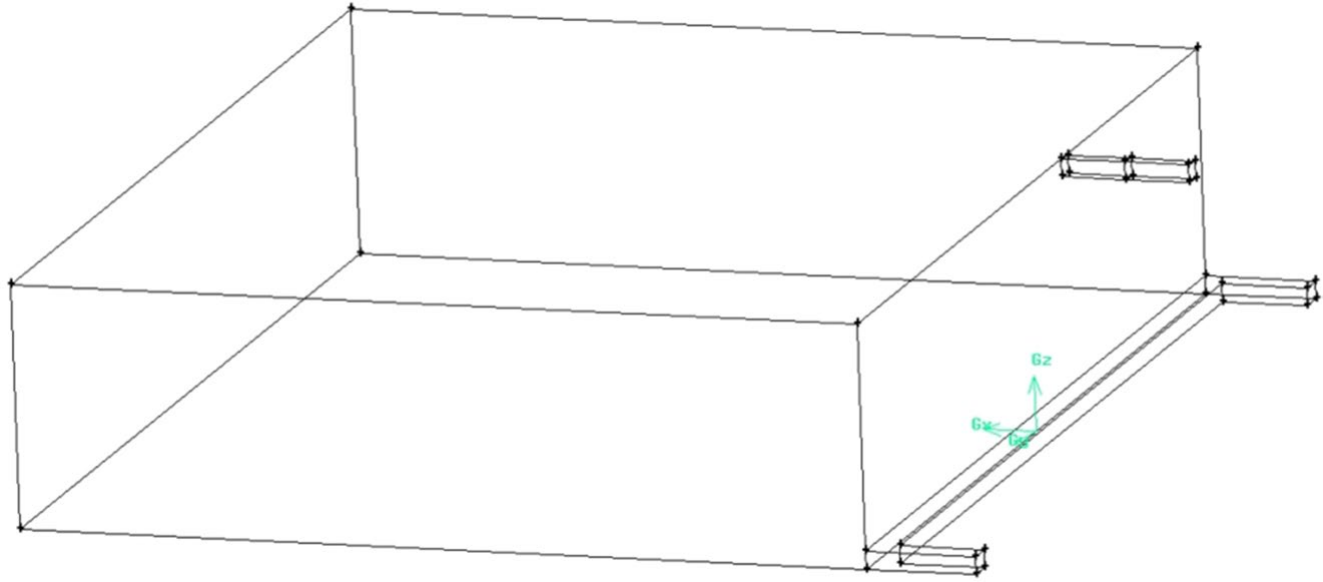
The geometric model of roof low roadway (FLUENT 16.0).

The geometric model of roof low roadway is established by “Goaf model and its gas distribution”, and drainage roadway is added. Assuming that the conditions and parameters remain unchanged, the model is shown in Fig. 8.

The upper corner gas concentration monitoring is set at the interface between working face and goaf, which is 20 cm away from the wall at the air return roadway. The coordinates of the monitoring points are set as (0, − 99.252, 14.253). Gas drainage detection face is set in roof low roadway.

**Figure 9:**
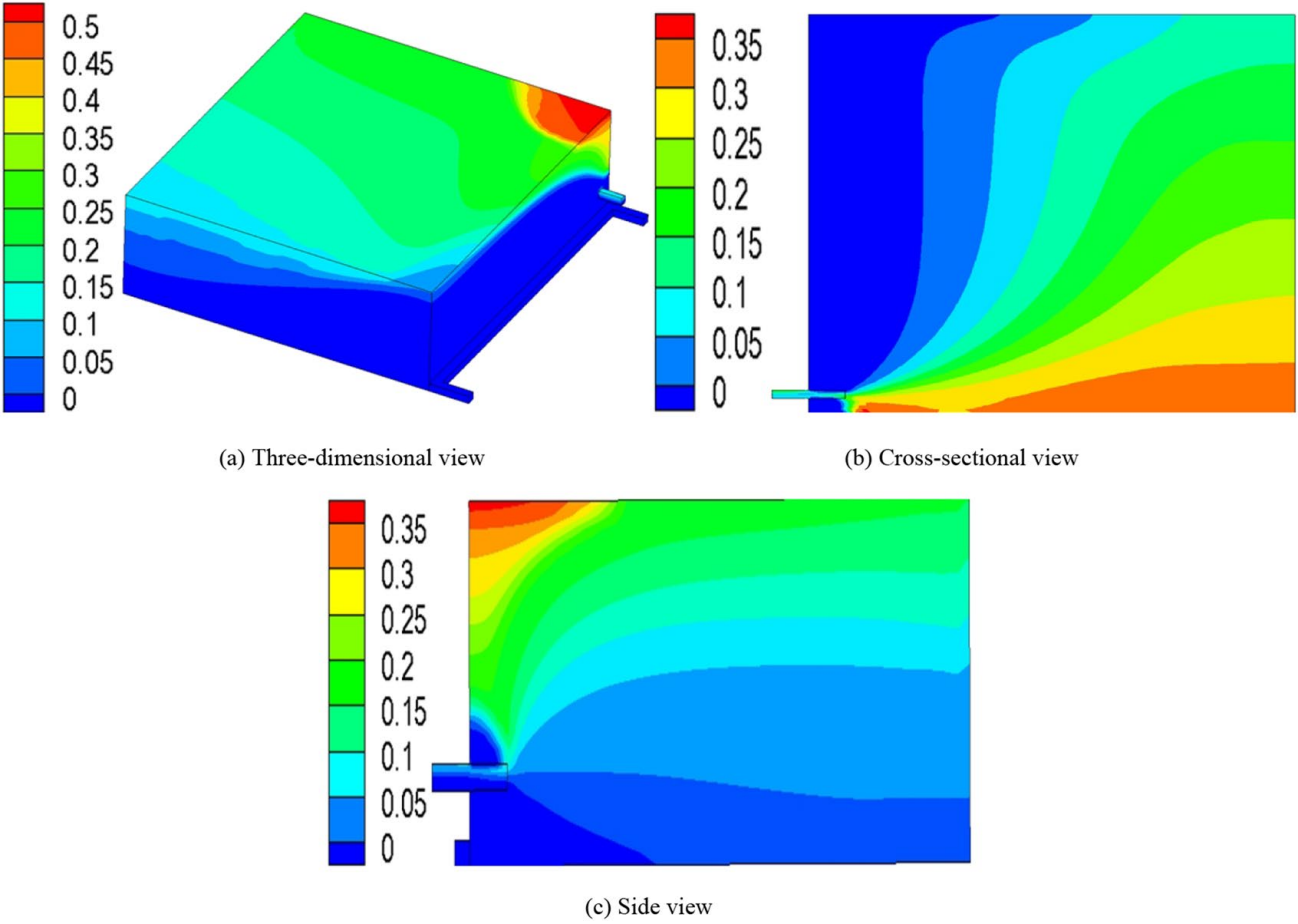
The drainage effect (FLUENT 16.0).

When the vertical distance between the roof low roadway and the roof of coal seam is 7 m, and the horizontal distance between the roof low roadway and the air return roadway is 5 m, the drainage effect is shown in Fig. 9a–c.

At the monitoring point, the gas concentration in the upper corner is 0.39% and gas drainage volume in the roof low roadway is 36.4 m^3^/min. The roof low roadway reduces the gas concentration around it.

## Response surface methodology

The factors influencing the effectiveness of gas concentration control in the upper corner include the vertical and horizontal distance of the roof low roadway. The central combination design has a wide range of applicability to the combination of factors and levels, and the regression equation obtained has a good fit with the actual results^49,50^. The central combination design is selected based on the two levels and two factors in this paper^51,52^.

Based on the central composite design principle, a 2-level experiment was designed with the vertical (X_1_) and horizontal (X_2_) distance of the roof low roadway as the influencing factors, and the gas concentration in the upper corner (Y_1_) and the gas drainage volume in the roof low roadway (Y_2_) as the response values, resulting in a response surface experiment with 13 sets of experimental points. The vertical and horizontal distances of the roof low roadway were investigated for optimal gas concentration in the upper corner and gas drainage volume in the roof low roadway. The regression equation is given in Eq. (3)

3 $$Y={\beta }_{0}+{\sum }_{i=1}^{n}{\beta }_{i}{X}_{i}+{\sum }_{i=1}^{n}{\beta }_{ii}{X}_{i}^{2}+{\sum }_{i<j}{\beta }_{ij}{X}_{i}{X}_{j}$$

where *Y* is the response value; *n* is the number of variables; *β*_0_ is a constant; *β*_i_ is the linear coefficient; *β*_ii_ is the quadratic term coefficient; and *β*_ij_ is the interaction coefficient.

The experimental factors and levels are determined according to the range of roof low roadway, as shown in Table 2.

**Table 2:** Factors and levels used for response surface of the central composite design.

Code number	Factors	Levels	
		Low	High
*X*_*1*_	Vertical distance (m)	5	9
*X*_*2*_	Horizontal distance (m)	3	7

Using Design-Expert software, the central composite design was selected and the influencing factors X_1_ and X_2_ and their high and low levels were input to obtain the response surface experimental design scheme, as shown in Table 3, and 13 sets of FLUENT simulations were done to derive the response values according to the scheme, as shown in Table 3.

**Table 3:** Comparison table of central composite design scheme and simulation response values.

Number	*X*_1_ (m)	*X*_2_ (m)	*Y*_1_ (%)	*Y*_2_ (m^3^/min)
1	9.82843	5	0.50	36.8
2	5	3	0.46	31.8
3	7	5	0.39	36.4
4	7	5	0.39	36.4
5	7	5	0.39	36.4
6	7	2.17157	0.425	34.2
7	5	7	0.51	33.7
8	9	3	0.46	34.4
9	4.17157	5	0.56	32.6
10	9	7	0.47	37.8
11	7	5	0.39	36.4
12	7	5	0.39	36.4
13	7	7.82843	0.41	36.1

A regression model was fitted to the experimental data and a multiple quadratic regression equation was obtained. The fitted equations for the gas concentration in the upper corner (Y_1_) and the gas drainage volume in the roof low roadway (Y_2_) are shown in Eqs. (4) and (5) respectively.

4 $${Y}_{1}=1.29422-0.24140{X}_{1}-0.015232{X}_{2}+0.017578{{X}_{1}}^{2}+0.00351563{{X}_{2}}^{2}-0.0025{X}_{1}{X}_{2}$$

5 $${Y}_{2}=15.02419+3.73373{X}_{1}+1.171794{X}_{2}-0.24375{{X}_{1}}^{2}-0.18750{{X}_{2}}^{2}+0.093750{X}_{1}{X}_{2}$$

The variance analysis of the gas concentration in the upper corner and the gas drainage volume in the roof low roadway is shown in Tables 4 and 5, respectively.

**Table 4:** Table of analysis of variance for quadratic model of gas concentration in upper corner.

Source	Degree of freedom	Mean square	F-valve	P-value	Significance
Model	5	0.00742	48.13	< 1 × 10^–4^	Extremely significant
*X*_1_	1	0.00195	12.64	0.0093	Significant
*X*_2_	1	0.00018	0.22	0.3059	Insignificant
*X*_1_^2^	1	0.034	223.11	< 1 × 10^–4^	Extremely significant
*X*_2_^2^	1	0.00138	8.92	0.0203	Significant
*X*_1_*X*_2_	1	0.0004	2.59	0.1517	Insignificant

**Table 5:** Table of analysis of variance for quadratic model of gas drainage volume in the roof low roadway.

Source	Degree of freedom	Mean square	F-valve	P-value	Significance
Model	5	7.57	37.18	< 1 × 10^–4^	Extremely significant
*X*_1_	1	19.97	98.05	< 1 × 10^–4^	Extremely significant
*X*_2_	1	7.97	39.15	0.0004	Significant
*X*_1_^2^	1	6.61	32.47	0.0007	Significant
*X*_2_^2^	1	3.91	19.21	0.0032	Significant
*X*_1_*X*_2_	1	0.56	2.76	0.1405	Insignificant

The P-value reflects the regression effect of the parameter^53–55^, P < 0.0001 means the regression effect of the factor is extremely significant, 0.0001 ≤ P ≤ 0.05 means the regression effect of the factor is significant, and P ≥ 0.05 means the regression effect of the factor is insignificant^56–58^. The Model F-value of 48.13 and 37.18 indicate that these two models are significant. There is only a 0.01% chance that an F-value this large could occur due to noise. The signal to noise ratio of the model is 19.289 and 17.654, both of which are greater than 4, indicates an adequate signal.

**Figure 10:**
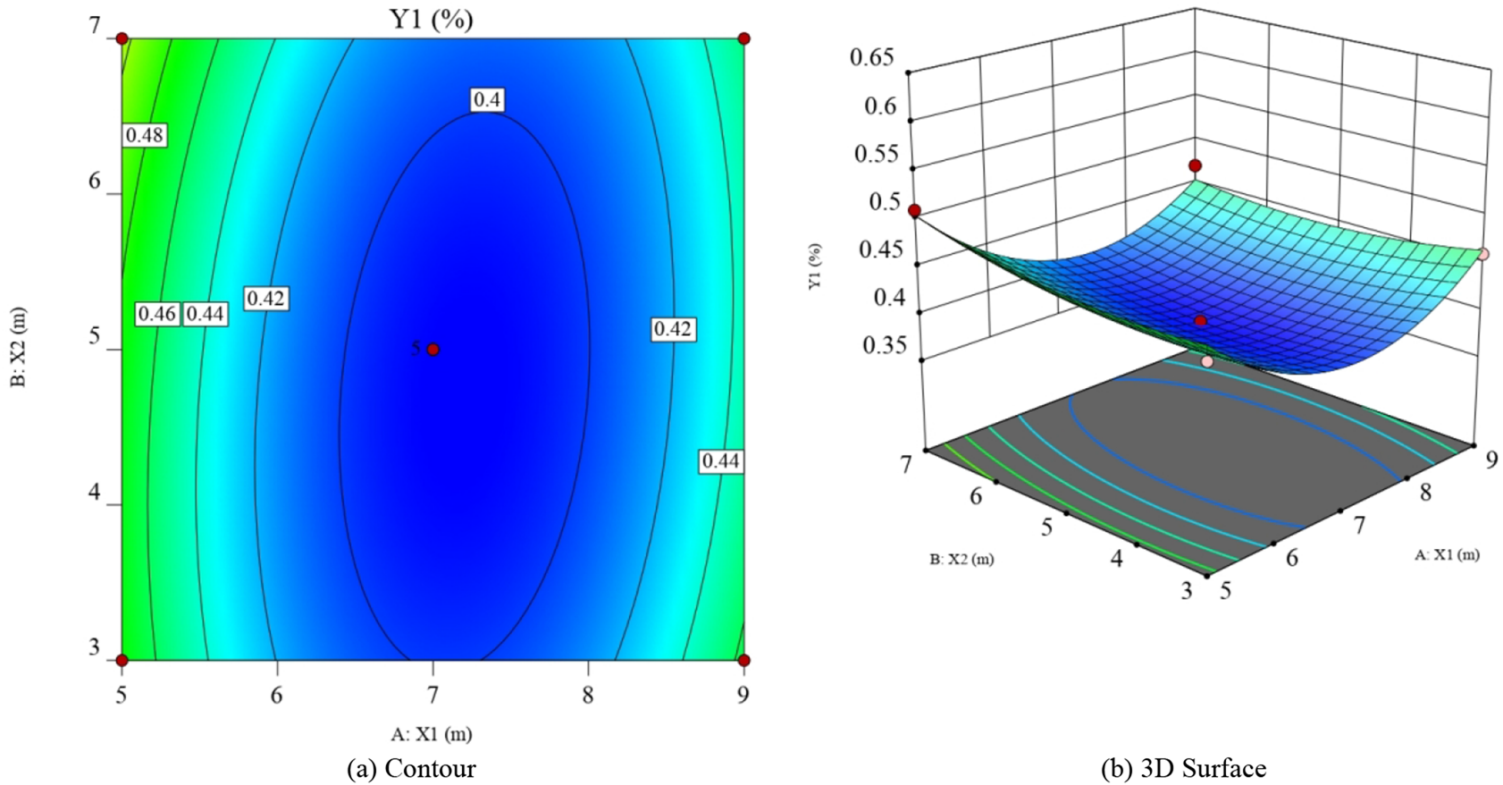
Response graph of the influence of various factors on the gas concentration in upper corner (Design-Expert 10).

**Figure 11:**
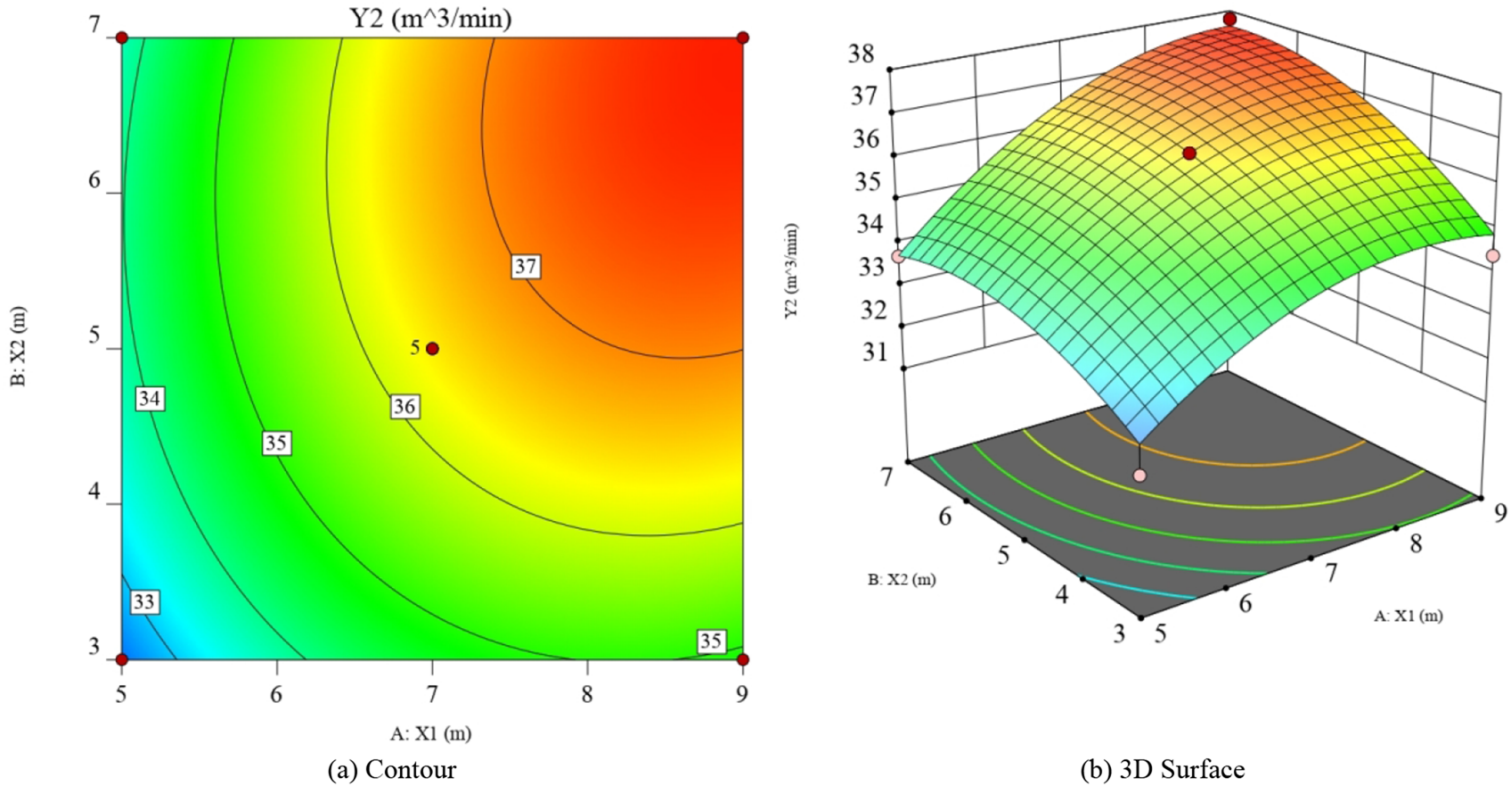
Response graph of the influence of various factors on the gas drainage volume in the roof low roadway (Design-Expert 10).

Response surface method analyses of the gas concentration upper corner (Y_1_) and the gas drainage volume in the roof low roadway (Y_2_) are shown in Figs. 10a,b and 11a,b.

With respect to the response value Y_1_, the interaction between vertical and horizontal distance was not significant, with the effect of vertical distance being significantly more significant than horizontal distance. From the response surface plot of Y_1_ and the positive quadratic term of Eq. (4), there is a minimum value of gas concentration in the upper corner. With respect to the response value Y_2_, the interaction between vertical and horizontal distance is not significant and the effect of both vertical and horizontal distance is more significant. From the response surface plot of Y_2_ and the negative quadratic term of Eq. (5), there is a maximum value of the gas drainage volume in the roof low roadway.

The response surface method can be used to predict the vertical and horizontal distances when the interaction between the gas concentration in the upper corner and the gas drainage volume in the roof low roadway is optimal, as shown in Table 6.

**Table 6:** Optimal results of response surface prediction.

*X*_1_ (m)	*X*_2_ (m)	Predictive value *Y*_1_ (%)	Predictive value *Y*_2_ (m^3/^min)
7.7	5.9	0.397	37.2

The optimal solution under the interaction of response surface prediction is selected as the parameter for numerical simulation experiment. It is obtained that gas concentration in the upper corner is 0.40%, the gas drainage volume in the roof low roadway is 37.4 m^3^/min, and the error from the predicted value is 0.75% and 0.53%, respectively. The predicted value is very close to the simulated value. The optimal position of the roof low roadway is: the vertical distance is 7.7 m, and the horizontal distance is 5.9 m.

## Effectiveness of gas control in the upper corner

After construction in accordance with the optimum position of the roof low roadway, the 15,106 working face was ventilated in a "U" pattern. During the normal mining period, the test was conducted for one month, and the average inlet air volume of the air inlet roadway was 3123 m^3^/min and the average return air volume of the air return roadway was 2160 m^3^/min. The gas concentration in the upper corner, the gas concentration in roof low roadway, and the daily mining distance were tested. Based on the test results, the gas drainage volume in roof low roadway was obtained.

**Figure 12:**
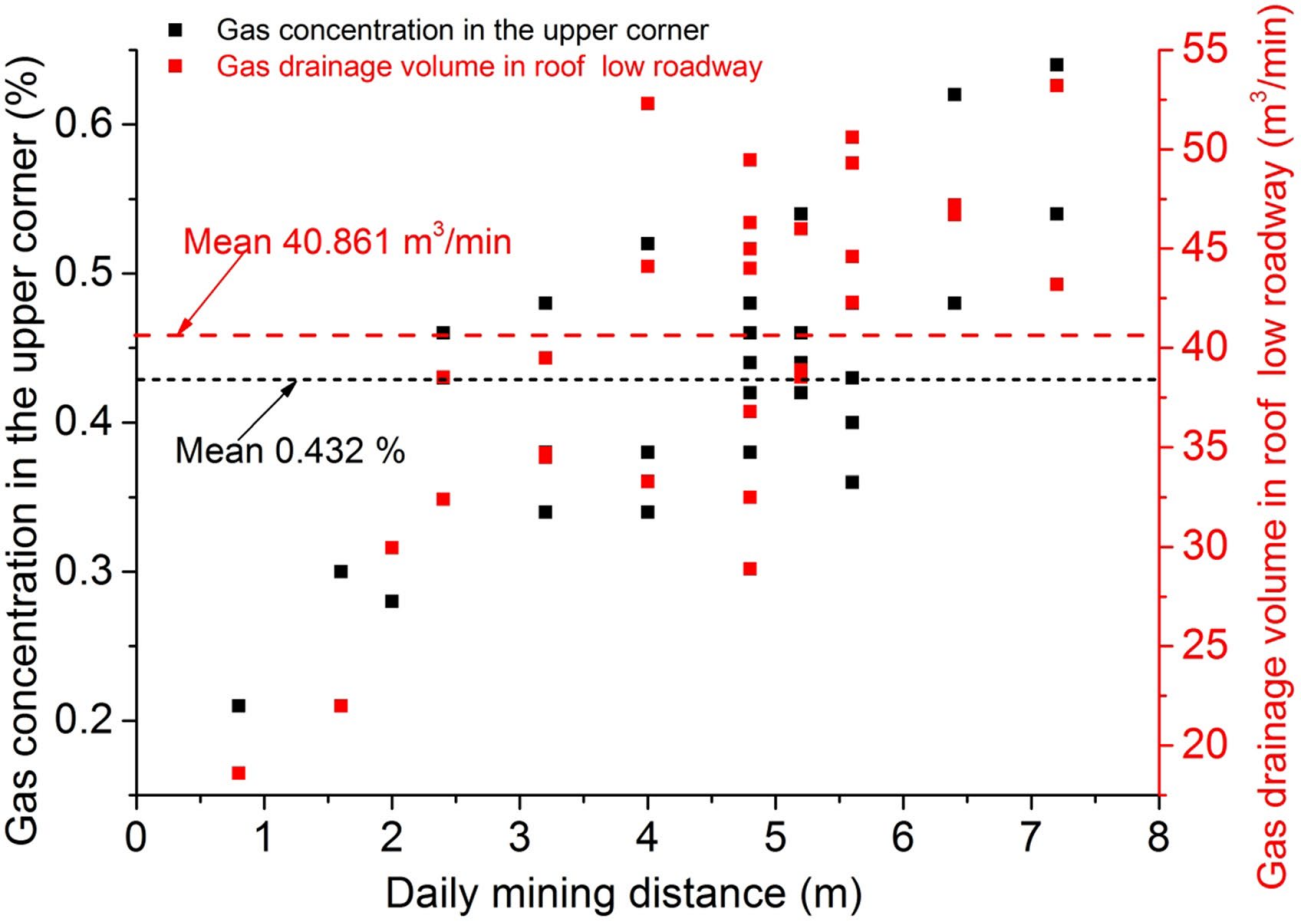
Relationship between daily mining distance and gas.

A scatter plot is made using the daily mining distance as the horizontal coordinate and the gas concentration in the upper corner and the gas drainage volume in roof low roadway as the vertical coordinates, as shown in Fig. 12.

The average daily mining of the working face during normal mining was calculated to be 4.493 m. The average gas concentration in the upper corner was 0.432%, an error of 8.8% compared to the simulation, and the average gas drainage volume in roof low roadway was 40.861 m^3^/min, an error of 9.8% compared to the simulation, which is a small error between the field test and the simulation. As can be seen in Fig. 11, the gas concentration in the upper corner and the gas drainage volume in roof low roadway show a certain trend of increase with the increase of the daily mining distance, indicating that reducing the mining speed can reduce the gas concentration in the upper corner to a certain extent.

The test results show that the design of roof low roadway, during normal mining, has no gas accumulation and effectively manages the gas accumulation problem in the upper corner.

## Conclusions

1. According to the fracture distribution law during the mining, the upper limit heights of the caving zone and the fractured zone are obtained as 18 m and 65 m, respectively. The off-bed fractures and through-bed fractures are mainly concentrated on the open-off cut side and mining side at the coal mining depth of 300 m. On this basis, the maximum porosity of the two is calculated to be 0.42 and 0.34, respectively, which conforms to the "O" shaped ring theory.
2. FLUENT numerical simulation shows the gas distribution law in the goaf, and the gas concentration on the side of the return air roadway is relatively high. After adding the roof low roadway, when the vertical distance and horizontal distance of the roof low roadway are 7 m and 5 m, respectively, the gas concentration in the upper corner and the gas drainage volume in roof low roadway are 0.39% and 36.4 m^3^/min, respectively, which reduces gas concentration on the side of the air return roadway.
3. Based on the calculated position range of the roof low roadway, taking the vertical distance and horizontal distance of the roof low roadway as the influencing factors, design the roof low roadway horizon experiment scheme according to the response surface method. FLUENT simulation experiment obtains the response value, predicting that the vertical and horizontal distances of the roof low roadway were 7.7 m and 5.9 m respectively when the interaction between the gas concentration in the upper corner and gas drainage volume in roof low roadway was optimal. Choose the optimal solution as the parameter to do the numerical simulation experiment, and the error between the experimental value and the predicted value is very small.
4. In the field application, the test shows that the average gas concentration in the upper corner and the average gas drainage volume in the roof low roadway are 0.432% and 40.861 m^3^/min respectively during the normal mining. The error of the experiment is less than 10%. The gas concentration in the upper corner and gas drainage volume in the roof low roadway show a certain increasing trend with the increase of the daily mining distance. The designed roof low roadway effectively controls the problem of gas accumulation in the upper corner.

## Data Availability

The primary data used to support the findings of this study are available from the corresponding author upon request.

## References

[1] Fang S (2021). The pressure relief protection effect of different strip widths, dip angles and pillar widths of an underside protective seam. PLoS ONE.

[2] Guo H, Yuan L, Shen B, Qu Q, Xue J (2012). Mining-induced strata stress changes, fractures and gas flow dynamics in multi-seam longwall mining. Int. J. Rock Mech. Min..

[3] Karacan CÖ, Olea RA (2013). Sequential Gaussian co-simulation of rate decline parameters of longwall gob gas ventholes. Int. J. Rock Mech. Min..

[4] Ting-xiang C, Shi-xuan Z, Yong-liang X, Zhi-jun Z (2011). Research on the coupling effects between stereo gas extraction and coal spontaneous combustion. Procedia Eng..

[5] Verma S, Chaudhari S (2016). Highlights from the literature on risk assessment techniques adopted in the mining industry: A review of past contributions, recent developments and future scope. Int. J. Min. Sci. Technol..

[6] Karacan CÖ, Ruiz FA, Cotè M, Phipps S (2011). Coal mine methane: A review of capture and utilization practices with benefits to mining safety and to greenhouse gas reduction. Int. J. Coal Geol..

[7] Li H (2020). The integrated drainage technique of directional high-level borehole of super large diameter on roof replacing roof extraction roadway: A case study of the underground Zhaozhuang Coal Mine. Energy Rep..

[8] Li T, Wu B, Lei B, Huang Q (2020). Study on air leakage and gas distribution in goaf of Y-type ventilation system. Energy Sources Part A Recov. Utilization Environ. Effects..

[9] Xie S-R (2012). Mechanism and experiment of substituting high drainage roadway with directional long drilling group to extract pressure-relief gas. J. Cent. South Univ..

[10] Gao H (2021). Reasonable arrangement of high-level orientation extraction boreholes of pressure relief gas in overlying strata under high-strength fully mechanized mining in low-gas-thick-coal seam. Shock. Vib..

[11] Yang F (2020). A comprehensive gas extraction system coupling high-level suction roadway and boreholes for gas disaster prevention in closely-spaced multiple coal seams. Energy Sources Part A Recov. Utilization Environ. Effects..

[12] Lu Y (2020). Numerical assessment of the influences of the coal spontaneous combustion on gas drainage methods optimization and its application. Combust. Sci. Technol..

[13] Lin B (2019). Significance of gas flow in anisotropic coal seams to underground gas drainage. J. Petrol Sci. Eng..

[14] Ye Q, Jia Z, Zheng C (2017). Study on hydraulic-controlled blasting technology for pressure relief and permeability improvement in a deep hole. J. Petrol. Sci. Eng..

[15] Skotniczny P (2014). Transient states in the flow of the air-methane mixture at the Longwall Outlet, Induced by a Sudden Methane Outflow. Arch. Min. Sci..

[16] Guo H, Li X, Cui H, Chen K, Zhang Y (2019). Effect of roof movement on gas flow in an extremely thick coal seam under fully mechanized sublevel caving mining conditions. Energy Sci. Eng..

[17] Li X, Wang C, Chen Y, Tang J, Li Y (2018). Design of gas drainage modes based on gas emission rate in a gob: A simulation study. Arab. J. Geosci..

[18] Wang Z, Ren T, Cheng Y (2017). Numerical investigations of methane flow characteristics on a longwall face Part II: Parametric studies. J. Nat. Gas Sci. Eng..

[19] Wang Z, Ren T, Cheng Y (2017). Numerical investigations of methane flow characteristics on a longwall face Part I: Methane emission and base model results. J. Nat. Gas Sci. Eng..

[20] Liu J, Gao J, Yang M, Wang D, Wang L (2019). Numerical simulation of parameters optimization for goaf gas boreholes. Adv. Civ. Eng..

[21] Wang G (2018). Porosity model and air leakage flow field simulation of goaf based on DEM-CFD. Arab. J. Geosci..

[22] Brodny J, Tutak M, John A (2018). Analysis of influence of types of rocks forming the goaf with caving on the physical parameters of air stream flowing through these gob and adjacent headings. Mechanics.

[23] Chen D, Nie W, Cai P, Liu Z (2018). The diffusion of dust in a fully-mechanized mining face with a mining height of 7 m and the application of wet dust-collecting nets. J. Clean. Prod..

[24] Chen X, Du Y, Wang L, Zhao S (2020). Evolution and application of airflow permeability characteristics of gob in roof cutting and pressure releasing mining method. Energy Sci. Eng..

[25] Zhuo H, Qin B, Qin Q, Su Z (2019). Modeling and simulation of coal spontaneous combustion in a gob of shallow buried coal seams. Process Saf. Environ. Prot..

[26] Deng Q-W, Liu X-H, Lu C, Lin Q-Z, Yu M-G (2013). Numerical simulation of spontaneous oxidation zone distribution in goaf under gas stereo drainage. Procedia Eng..

[27] Cao Z, He X, Wang E, Kong B (2018). Protection scope and gas extraction of the low-protective layer in a thin coal seam: lessons from the DaHe coalfield, China. Geosci. J..

[28] Zhang P (2020). Stability of a roadway below a coal seam under dynamic pressure: A case study of the 11123 Floor Gas Drainage Roadway of a Mine in Huainan, China. Adv. Civ. Eng..

[29] Li S-G, Shuang H-Q, Wang H-S, Song K-I, Liu L (2017). Extraction of pressurized gas in low air-conductivity coal seam using drainage roadway. Sustainability.

[30] Tang M, Jiang B, Zhang R, Yin Z, Dai G (2016). Numerical analysis on the influence of gas extraction on air leakage in the gob. J. Nat. Gas Sci. Eng..

[31] Zhang X-B, Yang M (2018). Determination of optimal extrication location of high extraction roadway of large-mining-height fully mechanized face. Adv. Civ. Eng..

[32] Hu G (2015). Adjacent seam pressure-relief gas drainage technique based on ground movement for initial mining phase of longwall face. Int. J. Rock Mech. Min..

[33] Huang B, Cheng Q, Zhao X, Kang C (2018). Hydraulic fracturing of hard top coal and roof for controlling gas during the initial mining stages in longwall top coal caving: A case study. J. Geophys. Eng..

[34] Gao M (2018). The location optimum and permeability-enhancing effect of a low-level shield rock roadway. Rock Mech. Rock Eng..

[35] Zheng C, Kizil M, Chen Z, Aminossadati S (2017). Effects of coal damage on permeability and gas drainage performance. Int. J. Min. Sci. Technol..

[36] Cui J (2021). Uncertainty analysis of mechanical dynamics by combining response surface method with signal decomposition technique. Mech. Syst. Signal Process..

[37] Darwish HW (2021). Response surface methodology for optimization of micellar-enhanced spectrofluorimetric method for assay of foretinib in bulk powder and human urine. Spectrochim. Acta Part A Mol. Biomol. Spectrosc..

[38] Karimifard S, Alavi Moghaddam MR (2019). Corrigendum to “Application of response surface methodology in physicochemical removal of dyes from wastewater: A critical review” [Sci. Total Environ. 640–641(2018) 772–797]. Sci. Total Environ..

[39] Wang W, Wang F, Lu F (2017). Microwave alkaline roasting-water dissolving process for germanium extraction from zinc oxide dust and its analysis by response surface methodology (RSM). Metall. Res. Technol..

[40] Shen B, Shi J, Barton N (2018). An approximate nonlinear modified Mohr–Coulomb shear strength criterion with critical state for intact rocks. J. Rock Mech. Geotech. Eng..

[41] Alshkane YM, Marshall AM, Stace LR (2017). Prediction of strength and deformability of an interlocked blocky rock mass using UDEC. J. Rock Mech. Geotech. Eng..

[42] Hauseux, P., Roubin, E. & Colliat, J.-B. The embedded finite element method (E-FEM) for multicracking of quasi-brittle materials. *Porous Rock Fracture Mechanics*, 177–196. 10.1016/b978-0-08-100781-5.00008-7 (2017).

[43] Zhang Z, Zhang R, Xie H, Gao M (2015). The relationships among stress, effective porosity and permeability of coal considering the distribution of natural fractures: Theoretical and experimental analyses. Environ. Earth Sci..

[44] Wang S, Li X, Wang D (2016). Void fraction distribution in overburden disturbed by longwall mining of coal. Environ. Earth Sci..

[45] Zhu H (2020). Study of the dynamic development law of overburden breakage on mining faces. Sci. Rep..

[46] Lu Y, Gadh R, Tautges TJ (2001). Feature based hex meshing methodology: Feature recognition and volume decomposition. Comput. Aided Des..

[47] Xiaofei Z (2020). Flow accelerated naphthenic acid corrosion during high acid crude oil refining. Eng. Fail. Anal..

[48] Lam WH, Robinson DJ, Hamill GA, Johnston HT (2012). An effective method for comparing the turbulence intensity from LDA measurements and CFD predictions within a ship propeller jet. Ocean Eng..

[49] Kashyap D, Das S, Kalita P (2021). Exploring the efficiency and pollutant emission of a dual fuel CI engine using biodiesel and producer gas: An optimization approach using response surface methodology. Sci. Total Environ..

[50] Medina MB, Resnik SL, Munitz MS (2021). Optimization of a rice cooking method using response surface methodology with desirability function approach to minimize pesticide concentration. Food Chem..

[51] Bezerra MA, Santelli RE, Oliveira EP, Villar LS, Escaleira LA (2008). Response surface methodology (RSM) as a tool for optimization in analytical chemistry. Talanta.

[52] Morris MD (2000). A class of three-level experimental designs for response surface modeling. Technometrics.

[53] Cao J (2021). Model-based strategy for nitrogen removal enhancement in full-scale wastewater treatment plants by GPS-X integrated with response surface methodology. Sci. Total Environ..

[54] Mhemed HA (2021). Corrigendum to “Gas adsorptive desulfurization of thiophene by spent coffee grounds-derived carbon optimized by response surface methodology: Isotherms and kinetics evaluation” [J. Environ. Chem. Eng. 8, 2020, 104036]. J. Environ. Chem. Eng..

[55] Zhang W (2021). Research on multivariate nonlinear regression model of specific energy of rock with laser drilling based on response surface methodology. Optics Commun..

[56] Teófilo RF, Ferreira MMC (2006). Quimiometria II: Planilhas eletrônicas para cálculos de planejamentos experimentais, um tutorial. Quim. Nova.

[57] Gilmour SG (2006). Response surface designs for experiments in bioprocessing. Biometrics.

[58] Box GEP (2018). Statistics as a catalyst to learning by scientific method Part II—A discussion. J. Qual. Technol..

